# Cost-effective horse breeding in the Republic of Bashkortostan, Russia

**DOI:** 10.14202/vetworld.2020.2039-2045

**Published:** 2020-10-01

**Authors:** Almir Askarov, Alfiya Kuznetsova, Rasul Gusmanov, Aigul Askarova, Vitaliy Kovshov

**Affiliations:** Federal State Budgetary Educational Establishment of Higher Education, Bashkir State Agrarian University, 50-Letia Octyabrya Str., 34, Ufa, Russian Federation

**Keywords:** break-even volume, efficiency, marginal income, productive droving horse-breeding

## Abstract

**Background and Aim:**

There is a lack of reliable data in agribusiness regarding the economic efficiency of horse breeding, and this limits its further development. The purpose of this study was to create rational parameters for the development of productive horse breeding as an effective agricultural business, in particular, in relation to farms.

**Materials and Methods:**

The methods of investigation used were induction and deduction, as well as analytical, statistical, and economic-mathematical analysis. We also used the dynamics of time series, CVP analysis, direct costing, and microeconomic analysis. Data were taken from the Russian Federation’s official statistics on animal husbandry as well as closed (commercial) data of agricultural enterprises from our study region.

**Results:**

Horse ownership in the Republic of Bashkortostan is higher than in the rest of Russia with about 9% of the total number of horses in Russia. We found that landowners need one hectare of arable land to ensure profit and that the highest economic income occurs on farms specializing in kumis production. The production of kumis under intensive farming is less profitable than with free-range horses kept in pastures. Family farms need a large amount of arable land with natural foliage to balance space and profit.

**Conclusion:**

Successful implementation of these parameters will make it possible to turn agriculture into successful horse breeding businesses. The expected volume of agricultural production may be approximately 9-11 thousand US dollars per employee.

## Introduction

The number of horses being kept in economically developed countries such as the United States, England, Germany, Italy, and France has increased in the last 50 years due to increases in the number of thoroughbreds used for horse racing, promenade saddling, equine tourism, horseback riding, equestrian plays, and horse competitions. In the United States, the equestrian industry in the year 2015 employed 4.6 million people [1].

Horses are typically bred for four industries: Commercial, productive (horse meat and milk), sports and leisure horse breeding, and pedigree (stud farm) horse breeding. Some researchers also designate some breeding as applied horse breeding, in which horses are bred for the production of raw materials for the pharmaceutical and processing industries, and some breeding as hippotherapy, in which horses are bred for use as a part of a post-discharge adjustment period for the treatment of complex neurological and mental diseases, especially in children [2,3].

Productive horse breeding is of great importance for the Russian Federation. The use of horses in Russia is more economically advantageous for small farms than the use of farm machinery [4]. Horses can also be used for equestrian leisure, mounted police, and border guard service in remote areas. The horse breeding industry has the potential to be profitable in Russia, however, a significant number of the 68 stud horse farms and 123 reproduction breeding units in the country are unprofitable [5,6].

This unprofitability needs to be improved, but modern authors focus on technological, veterinary, and zootechnical aspects of the horse breeding industry [7,8]. Improving economic efficiency, kumis and horse meat production, cost accounting, and herd reproduction are not fully covered. Kumis has long been considered not only a valuable food product but also a drink with high healing properties. In Bashkiria, Kazakhstan, Uzbekistan, Kyrgyzstan, Tatarstan, Kalmykia, and Yakutia, kumis is a folk remedy and a common drink. Kumis is a fermented dairy product made mainly from mare’s milk and has been successfully used to treat tuberculosis patients.

It is our opinion that both scientists and the public should focus their attention on this promising branch of animal breeding, particularly in steppe zones where meat and dairy horse breeding historically developed. The aim of this study was to develop recommendations that can be used to improve the economic efficiency of horse breeding in Russia. To achieve this goal, we completed the following tasks:

- Studied current state and development trends in the productive horse breeding industry, to justify the economic importance of productive horse breeding as a high-margin agribusiness
- Developed scientifically based proposals to improve economic efficiency, sustainability, and the competitiveness of productive horse breeding
- Justified the main directions of horse breeding development under current conditions.

## Materials and Methods

### Ethical approval

The study was conducted in accordance with the ethical principles approved by the Ethics Committee, Federal State Budgetary Educational Institution of Higher Professional Education “Bashkir State Agrarian University” (Protocol No. 8 of 28.06.2019).

### The object of the study

We selected the Republic of Bashkortostan as the focus of our research, as the region ranks second in the number of horses owned in the Russian Federation. The horse population in the region consists of 11% of the total number of horses in the country [4]. More than 3 tons of kumis is produced in the Republic of Bashkortostan, which is more than 45% of the total volume produced in Russia. A total of 11.5 thousand tons of horse meat are produced in the republic**.** Of these, more than 60% of kumis and 30% of horse meat are produced in five of the 54 municipal districts, which are geographically located in the Trans-Ural and Cis-Ural steppe zones of the republic [9,10]. Productive horse breeding in these areas is highly profitable both for farm enterprises with several hundred horses and for those with only 10-50 mares. The productive horse breeding industry has clean-cut zoning features. The object of this study was the farms of the Abzelilovsky municipal district, since they contain the largest number of horses with 13% of the total number of horses in the Republic of Bashkortostan.

### Research methodology

We used margin analysis and statistical data analysis to examine consolidated annual reports from agricultural organizations in the Republic of Bashkortostan from 2014 to 2017, as well as reports as from some farms located in our study region. When developing economic models of horse farms, standard calculation methods were used.

Margin income per one hectare of arable land was used as the main criterion for assessing the market efficiency of the industry and as the benchmark for the enterprise’s future sustainability. The profit used to describe and evaluate the current state of a commercial organization, as well as of a separate product during the reporting period, cannot be a criterion [11]. This is due to a significant differentiation of the level of purchase prices at the same level of cost.

When calculating margin income, the total costs are divided into fixed/semi-fixed and variable/semi-variable costs. We carefully studied the costs and added the following items into our calculations:

- In crop production: Wages plus social payments, seeds and planting material, mineral and organic fertilizers, and costs for fuel and lubricants
- In animal breeding: Wages plus payments and feed costs.

Fixed costs included electricity and fuel, depreciation, spare parts, and other patching materials for repairing fixed assets, other expenditures including material costs as well as payment for services and works performed by outside organizations.

### Statistical analysis

Statistical observation was based on collected quantitative data from the Russian Federation’s official statistics on animal husbandry. Statistical material was processed and presented in tables, charts, and graphs. Statistical and mathematical data processing was completed with Statistica 6 (Statsoft, Tulsa, USA) and Microsoft Excel (IBM corp. USA).

## Results

**Figure-1:**
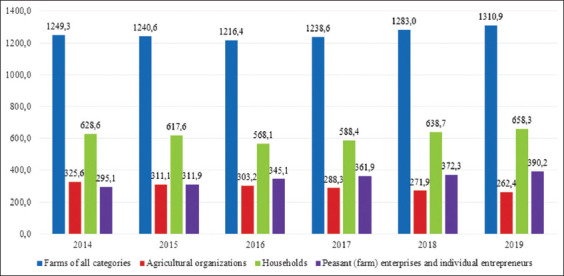
Number of horses on farms of all categories of the Republic of Bashkortostan for the years 2014-2019 (thousand heads).

The Republic of Bashkortostan has had the highest population of horses in for a long period of time. Over the period from 2014 to 2019, horse stocks in large agricultural organizations declined steadily by 20%. The number of horses in peasant (farmer) households increased steadily by 32% during this period, while personal households increased by almost 5% (Figure-1).

The data presented in Figure-1 show that the total number of horses in agricultural organizations decreased by 10.8%, while the number held by private farms increased by a factor of 2.1 from 23.2 to 47.7 thousand heads. On peasant farms, the number of horses increased to 36.3 thousand heads in 2018. In 2014, 30.5% of the total number of horses in the region belonged to agricultural organizations, while individual farms held 51.1% of horses, 18.4% of which were on peasant farms. In 2018, agricultural organizations owned 25.2% of horses, while private farms held 42.5% and peasant farms held 32.3%.

**Figure-2:**
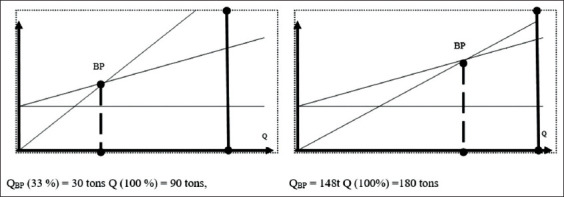
Break-even kumis output volume when horses are kept in herds and on pastures.

As reported in Table-1, the highest amount of marginal income or payment to cover fixed costs received from one hectare of arable land belongs to productive horse breeding. Kumis farms, where horses are kept in industrialized herds, have higher economic indicators compared with farms where the horses are kept in pastures (Table-2). Only 33% of production capacity is needed to reach a break-even level of production when horses are kept in stalls, as compared to kumis farms where horses are kept in pastures, which needs more than 80% of production capacity to break even (Figure-2).

**Table 1:** Economic characteristics of production types (industries) of the pilot area.

Type of production (industry)	Yield capacity, cwt/ha (productivity, cwt per head)	Production price US $/cwt	Production cost US $/cwt		Profit, US $/cwt	Margin return (US $) on	
	
			Full	Truncated		1cwt	1 ha of arable land*
1	2	3	4	5	6	7	8
Cows							
Milk	26.6	23.4	20.2	14.1	3.3	6.1	58.3
Gain	1.6	109.1	142.8	100.0	−33.8	9.1	
Broodmares							
Kumis	3.5	166.7	116.7	81.7	50.0	85.0	108.3
Gain	1.0	175.0	56.7	39.7	118.3	135.3	
Ewes							
Wool	0.02	20.0	14.2	9.9	5.8	10.1	66.7
Gain	0.3	166.7	77.5	54.3	89.2	112.4	

* When determining the area of arable land required obtaining feed stuff per a female animal, we used general feeding standards for farm animals, taking into account the average land productivity of the region

**Table 2:** Main economic indicators on farms with different ways of horse keeping.

Indicator	Type of horse keeping	

	In herds	On pastures
Milk mares population, heads	150	150
Kumis season duration	170	365
Milk yield per a mare, kg	600	1200
Total kumis output, tons	90	180
Aggregate cost, thousand US $	106.3	470.0
Level of profitability, %	41.1	6.4
Truncated cost, thousand US $	79.8	329.0
Production cost, thousand US $	150.0	500.0
Marginal income, US $/cwt	88.7	95.0
Break-even kumis output volume, tons	30	148
Minimum number of mares to obtain break-even kumis output volume, head	50	124

The exchange rate of cost indicators in the table is 60 RUB/US $

Structural changes have led to a sharp drop in agricultural sector employment. In this regard, a significant part of the rural population has become migrant workers, since they cannot find work near their homes. This problem could be solved by creating favorable conditions for the development of small- and medium-sized businesses focused on processing, marketing, and service organizations in rural areas.

The economic models that we have developed in this study could be used to contribute to the realization of this goal (Table-3).

**Table 3:** Basic parameters of horse peasant farms.

Parameters	Variants		

	I	II	III
Number of people able to work, person.	2	3	6
Number of animals, heads			
Stud getter	1	1	2
Mares	10	25	50
Foals	8	20	40
Young horses (12-24 months)	16	40	80
Working horses	2	4	5
Cows	3	2	1
Baby beefs	6	4	2
Farmland area, ha	97	134	394
Including: Arable land	17	21	67

We have organized private and public entities holding horses by size. Variant I includes family farms that have 10 mares and three cows (including young horses). Variant II includes 20 mares and five cows. Variant III includes farms that hold 50 mares and one cow (including young horses). Variant I farms need 97 hectares of land for farming, of which 80 hectares are natural forage land, while Variant III needs 394 hectares of farmland with 327 hectares of natural forage land. The annual farm output for Variant I is 17.2 thousand US dollars. The annual farm output for Variant II is 33.0 thousand US dollars, while Variant III is 60.6 thousand US dollars (Table-4).

**Table 4:** Economic results of horse peasant farms.

Parameters	Variants		

	I	II	III
Product yield, cwt: Including: Horse meat (body weight)	10.0	25.0	50.0
Kumis	60.0	150.0	300.0
Milk	105	70	35
Beef (body weight)	11.4	7.6	3.8
Production cost, thousand US $ totally	17.2	33.0	60.6
In particular per one employee	8.6	11.0	10.1
Output per 1 ha of farmland, thousand US $	0.2	0.2	0.2
Output per 1 work hour, US $	4.5	5.4	5.5
Share of horse breeding in output, %	74	92	98

The exchange rate of cost indicators in the table is 60 RUB/US $.2. To maintain cost indicators comparability, the prices established in the pilot region by 2015 were used everywhere

According to our models, production activity profitability is quite comparable to the profitability of existing peasant farms specializing in other fields. The average peasant farm in the study region should have an area of arable land per farm of about 80 hectares, with 8-10 cows, an average of three workers, and a volume of agricultural production of about 9-11 thousand US dollars per employee.

## Discussion

Many scientific papers are primarily devoted to the study of horse breeding from the point of view of the use of horses in other areas [7,8,12,13]. Thus, serious attention is paid to the issues of genetics management, nutrition, technology, veterinary medicine, breeding, and ecology [14-22]. In this regard, it is almost impossible to compare productive horse breeding using economic parameters. One reason for this is the specific character of productive horse breeding. This is especially true for droving horse breeding, which is limited in many countries due to limited natural forage lands. A second reason for this is western farmers, especially from economically developed countries, who are most interested in product quality, particularly from a veterinary viewpoint [15,22].

According to our research, the Russia-wide trend of reducing the number of horses is also typical for the pilot region. Some of the reasons for this reduction include the migration of people from villages to cities, land labor predominance in agriculture, high resource prices, and low performance motivation in the agricultural sector. Due to the low cost of horse breeding products and current prices, horse breeding farms could earn a significant income. The profitability level of productive horse breeding in most regions provides expanded reproduction of the industry [6]. All the necessary prerequisites for the successful development of productive horse breeding exist in modern Russia. Despite the scientific and practical experience of many generations of horse breeders and national traditions, the horse breeding industry in Russia is poorly developed.

There are two main technologies used in productive horse breeding: Droving horse breeding and horse grazing. Advantages of these technologies include their minimal amount of required labor and their material resource use [4]. An important advantage of droving horse breeding is the maximum possible use of natural forage lands, which occupies half of the agricultural land in the country. By increasing the use of natural foraging lands, meat and dairy horse breeding can increase, which would lead to the sustainable development of the entire agricultural economy of the country [23,24].

There is a lack of theory-based and practice proven data on the effectiveness of agricultural companies specializing in horse breeding products such as kumis. According to experts, the demand for this product in Russia has been satisfied by under 10% of consumers [25,26]. Since horse milk is widely used in TB laboratories, efficient horse production and profit are possible, although the profitability depends on several factors [25]. To supply medical institutions with the necessary amount of kumis and to satisfy the needs for mare’s milk for foals in the future, it is necessary to develop and expand the existing kumis production and to create new kumis farms [6,26]. The most important factor, in our opinion, is the comparative assessment of production efficiency using the profitability coefficient or profitability per unit of output, profit margin [27], or fixed cost payments [28]. Tables-3 and 4 show the difference between the price of a product and the cost of a unit of goods, determined in accordance with the direct costs system. This criterion shows the contribution of the production unit to the profits of the enterprise. Table-4 depicts the total amount of revenue from sales of products per unit of accepted unit of measurement and the sum of the variable production costs of the same production volume (e.g., one ha, one head, and one working hour).

Market research [6] has shown that the market niche for horse breeding production is not entirely filled. Additional growth could occur in the production of kumis, for example, which is famous for its antibacterial properties and is used to treat tuberculosis, exhaustion, and anemia. Kumis is also used in cosmetology to treat skin diseases. Since mare’s milk is close in composition to human milk, feeding mare’s milk to human babies produces positive results, as it does not cause allergic reactions. Mare’s milk can even be used to produce various dairy products and for dietary nutrition, which has become very popular. The production of kumis requires much less of their production capacity (33%) to reach the break-even level of production, as compared to kumis farms with stable grazed horses (more than 80%).

The models of horse breeding farms we propose in our research are based on the maximum use of natural forage lands as the source of the cheapest feed. Variant I (10 mares and 3 cows) requires 97 hectares of farmland, including 80 hectares of natural forage land. Variant III (50 mares and 1 cow) needs 394 hectares of farmland, including 327 ha of natural forage land. These amounts guarantee effective farm management and healthy products. Spring-summer and autumn-winter pastures are important for grazing health and night grazing is a must for effective feeding. This allows for the release of significant areas of arable land, which can be used to increase the production of strong and valuable wheat varieties, which is especially important for the farms in the steppes and which contributes to the increased efficiency and profitability of crop industries.

The main cost items in droving horse breeding are wages and overhead costs. Feed costs are low, since horses kept in pastures cover up to 80% of their dietary needs from their summer and winter pastures. Significant labor costs are also not required. In this regard, droving horse breeding is equal to or better than other sectors of pastoral animal breeding in terms of labor productivity. These low costs allow this industry to be highly profitable in most areas of its distribution, but there is still the problem of seasonal production variability and cannot provide the population with kumis during the winter.

The increase in the number of horses may be limited by the insufficient size of natural pastures characteristic of the steppe zone (Table-4). Overloaded pastures with a large grazing number of grazing horses causes the grass to degrade and the productivity of these pastures to decrease. The lack of grazing feed also leads to a sharp increase in the cost of animal products, including horse products. This fact will negatively affect the economic efficiency of all agricultural production [6,25,26]. The average peasant economy of the pilot region (arable land per farm is about 80 hectares, the number of cows is 8-10 heads, and the average number of workers is three people) is able to produce agricultural products per worker of about 2-10.8 thousand US dollars.

Steppe regions are favorable for this kind of horse breeding [23-25]. At the district and zonal levels, the cost ratio is largely determined by natural and climatic conditions. The profitability of production activity, according to the presented models, is quite comparable with the profitability of existing peasant farms in other industries.

Around the world, there is a transition from traditional agriculture with a high proportion of manual labor to industrial agriculture, making it necessary to diversify agricultural enterprises and create new opportunities to preserve livelihoods in the country [29], including through the development of horse breeding. Horse breeding was used successfully in Brazil to preserve rural areas and traditional rural communities [8].

## Conclusion

We conclude that the production of kumis is profitable for the equine industry in the Republic of Bashkortostan, due to the presence of natural foliage for grazing, which reduces costs and increases profits. We found that the number of horses in the study area decreased over time and that horse ownership changed from agricultural organizations to private farms (companies or farmers). We also found that landowners need one hectare of arable land to ensure profit and that the highest economic income occurs on farms specializing in kumis production. The production of kumis under intensive farming is less profitable than with free-range horses kept in pastures. Family farms need a large amount of arable land with natural foliage to balance space and profit. With proper management, horse farms can be profitable.

To supply large cities with kumis year-round and to provide industrial centers and medical institutions with valuable medicinal product, the number of horses should increase by a factor of four, and the level of specialization should be in the range of 74-98%, which will increase the industry’s profitability from 6 to 40% or more. Agricultural producers from regions with similar climatic conditions could use these results to further develop the horse breeding industry, regardless of which country or region they live in.

## Authors’ Contributions

AA: Coordination of the experiments and generalization of results of scientific and economic experiments, primary author, and general manager. AK: Generalization of the literature review and organization of discussion of the results of scientific and economic experiments. RG: Collected the results of this test. AiA: Collected the results of this test and wrote the manuscript. VK: Collected the results of this test and wrote the manuscript. All authors read and approved the final manuscript.
